# Health professionals knowledge of telemedicine and its associated factors working at private hospitals in resource-limited settings

**DOI:** 10.3389/fdgth.2022.976566

**Published:** 2022-09-16

**Authors:** Sisay Maru Wubante, Masresha Derese Tegegne

**Affiliations:** Department of Health Informatics, Institute of Public Health, College of Medicine and Health Sciences, University of Gondar, Gondar, Ethiopia

**Keywords:** knowledge, telemedicine, health professionals, factors, Ethiopia

## Abstract

**Introduction:**

The appropriate implementation of telemedicine in the healthcare system has the potential to overcome global problems such as accessibility and quality healthcare services. Thus assessing the knowledge of health professionals before the actual adoption of telemedicine is considered a prominent solution to the problems.

**Objective:**

This study aimed to assess healthcare professionals' knowledge of telemedicine and its associated factors at private hospitals in low-resource settings.

**Method:**

An institution-based cross-sectional study was conducted among 423 health professionals at private hospitals in low-income settings in Ethiopia, from March to April 2021. Data collection was performed by pretested and self-administered questionnaires. This study employed statistical packages for social sciences software. This study employed multivariable logistic regression to determine dependent and independent variables associated with adjusted odd ratio and 95% CI.

**Result:**

in this study about 65.8% of health professionals have good knowledge on Telemedicine .Computer literacy (AOR = 2.9; 95% CI: 1.8, 4.6), computer training (AOR = 2.0; 95% CI: 1.2, 3.3), Internet availability at workplace (AOR = 2.1; 95%CI: 1.3, 3.4), had private laptop (AOR = 1.7; 95% CI: 1.1, 2.9) were significantly associated with knowledge.

**Conclusion and recommendation:**

In general health professionals had good knowledge of Telemedicine. Inclusive packages of capacity by training among health providers are fundamental for the successful implementation of telemedicine.

## Introductions

Even though the field of digital technology rapidly moving forward in our worldwide, its utilization in the practice of medicine and patient care has remained suboptimal (1).

Compared to other sectors healthcare sectors are ineffective in the utilization of digital technologies. Telemedicine is the use of information technology to endorse and enable long-distance patient care, the upkeep of patient health records, and the offers patient and professional health (2). According to the World health organization (WHO), Telemedicine is an affordable use of information technology to support health and health-related fields such as healthcare service, medical education, and public health surveillance (3). Today Telemedicine is the fastest growing sector of health care, and much of this growth can be attributed to the benefits of telemedicine, such as shorter wait times, reduced travel, and fewer missed appointments (4). it has been recognized as a means to improve remote access, quality, and efficiency of care (5). Telemedicine employs a variety of technologies, including smartphones, computer tablets, mobile applications, and video conferencing, to enable health care providers to virtually evaluate, diagnose, monitor, treat, and educate patients (6). Although uses of various technologies to assist patients and have historically been used to provide care to patients is low in resource-constrained settings (7, 8). Thus, World Health Organization agreed on the contribution of telemedicine and approved that appropriate usage of this technology can support the healthcare sector in many countries, and considerably expand the quality of well-being care facilities, especially for low-income, and medically underserved communities (9).

Many possible explanations for why this technology adoption remains difficult include the knowledge and understanding of the principles, learning, perception, and workplace conditions of the concerned professionals (10).

A study conducted in Saudi Arabia on health professionals' knowledge of telemedicine stated that 46.1%of health professionals had good knowledge (11).

A study carried out in Ethiopia's public facilities on health professionals’ knowledge of telemedicine found that 37.6%of respondents had enough knowledge (12).

Therefore the users' knowledge of the technology is an important issue that should be considered before beginning a telemedicine program (13).

There are multiple explanations for how telemedicine system advancement and incorporation remain complicated in this area of the globe. Any innovative technology's effectiveness and future growth are largely influenced by factors such as user knowledge and understanding of the current principle, abilities needed for implementation, and a workplace atmosphere enabling technology acceptance (8, 14–16). Thus, for telemedicine to be properly implemented in the Ethiopian healthcare industry, research to demonstrate telemedicine understanding among health providers is needed.

As a result, recognizing health workers' telemedicine insight as well as attributes has been essential for emphasizing action to enhance telemedicine system adoption. Given this context, promoting the rewards of telemedicine platforms, policies, and approaches is necessary to boost affordable healthcare for disadvantaged communities, as well as the standard of healthcare and the ability to share legitimate healthcare information for scientific proof-of-concept among healthcare workers. Nevertheless, the incorporation of a telemedicine system is essential; according to the researcher's knowledge, few studies on the Knowledge of telemedicine systems are among Ethiopian health workers working in non-governmental hospitals.

Therefore this study aimed to assess health professionals' knowledge of telemedicine and its associated factors at private hospitals in northwest Ethiopia.

## Method

### Study period and design

From March 3 to April 7, 2021, and institutional cross-sectional study with a quantitative approach was conducted at private hospitals in resource-limited settings in northwest Ethiopia.

### Study population and sample size determination

The subjects of the study were health professionals from private hospitals in low-resource settings. The sample size was determined using a single population proportion equation, assuming a 50% response rate and a 10% non-response rate because no previous research had been conducted. Subsequently, a required sample size of 423 was acquired. The health professionals with less than six months of clinical practice and experience were excluded from the study. Respondents were chosen from private hospitals by simple random sampling technique.

### Data collection tool and quality assurance

Data were collected by using A structured self-administered questionnaire designed for the study. For linguistic uniformity, the questionnaire was first written in English and then translated into Amharic. Four health informatics professionals' supervisors were recruited and eight health information technicians were data collectors. The one-day training was given to supervisors and data collectors on the objective of the study, data collection process, how to approach respondents and quality of the data, and safeguarding of information. The 10% of respondents were assessed before the actual data collection.

### Statistical analysis

The data entry and analysis were done by using Epi info 7.2 version and SPSS version 20 respectively. Descriptive statistics from sociodemographic characteristics, organization factors, and technical factors were computed .this presented in the form of a table, graph, and text. We used binary logistic regression to analyze the association of independent variables with the outcome variables. Variables having statistically significant association with the outcome variable (*p* < 0.2), in the bi-variable analysis, were included in multivariable logistic regression analysis for controlling the effect of the confounder. Variables' significant association was determined based on adjusted odd ration (AOR), with 95% Cl and variables with (*p* < 0.05) were considered as determinant factors for health professional knowledge on Telemedicine.

### Measurement

#### Knowledge

The level of knowledge of respondents about telemedicine was assessed using 18 questions. This research used the average score of 9 (50%) from the 18 questions as a cutoff point to determine the level of telemedicine knowledge. The mean knowledge score of less than nine was labeled as poor knowledge of telemedicine, and the more than average score of nine was labeled as good knowledge of telemedicine (12).

## Result

### Socio-demographic characteristics of study subjects

As shown in Table 1, about 423 health professionals were approached for this study, with 410 responding at a 96.9 percent response rate. More than half 226 (55.1%). of respondents were males. The majority of study participants (44.6%) were between the ages of 25 and 29 (Table 1).

**Table 1:** Socio-demographic characteristics of respondents working at private hospitals limited resource settings Ethiopia 2021.

Variables	Categories	Frequency (N)	Percentage (%)
Sex	Male	226	55.1
	Female	184	44.9
Age	20–24	29	7.1
	25–29	183	44.6
	30–34	128	31.2
	≥35	70	17.1
Professions	physician	90	22.0
	clinical Nurse	165	40.2
	midwifery	40	9.8
	pharmacy	46	11.2
	Medical laboratory	57	13.9
	Other	12	2.9
Work experiences	<2	66	16.1
	2–3	58	14.1
	4–5	67	16.3
	>5	219	53.4
Educational status	Diploma	82	20.0
	Degree	202	49.3
	Masters and above	126	30.7

### Institutional factors

Roughly 245 (59.8%) of study subjects had access to a computer at the workplace. Regarding internet connectivity workplace, nearly two-thirds of 251 (61.2%) of the study subjects have available to it (Table 2).

**Table 2:** Institutional factors respondents’ knowledge of telemedicine at private hospitals limited resource settings Ethiopia 2021.

Variables	Groups	Occurrence (*N*)	Percentage (%)
Computer access in the workplace	Yes	245	59.8
	No	165	40.2
Internet access at the workplace	Yes	251	61.2
	No	159	38.8
Accessible IT care	Yes	252	61.5
	No	158	38.5
Computer Training	Yes	168	41.0
	No	242	59.0
Backup power generator	Yes	334	81.5
	No	76	18.5

### Knowledge of study subjects on telemedicine

**Figure 1:**
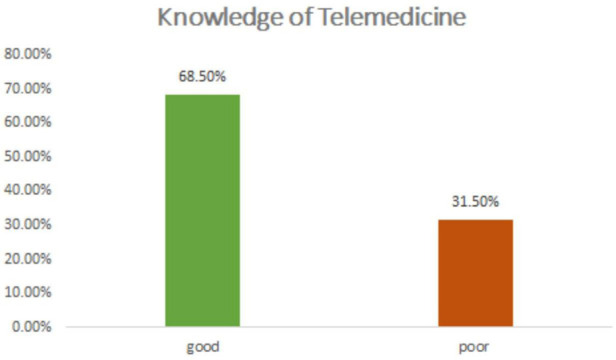
Study subjects knowledge of telemedicine at private hospitals norwest Ethiopia 2021.

As shown in Figure 1 more than two-thirds of 281 (68.5%) of the respondents had good knowledge.

## Factors affecting health professionals’ telemedicine knowledge

In Multiple logistic regression variables these as own personal laptop, internet connectivity at the workplace, computer literacy, and computer training has been associated with knowledge of Telemedicine, as shown (Table 3). Study subjects who had laptops were about 1.7 times more likely (AOR = 1.7; 95%CI: [1.1, 2.9]) to have good knowledge of Telemedicine than study participants who did not own a laptop.

**Table 3:** Bivariable and multi-variable regression for determinants for telemedicine knowledge among study subjects at private hospitals north west Ethiopia 2021.

Variables	Knowledge		Crude OR (95% CI)	Adjusted OR (95% CI)	*p*-value
	Good	Poor			
**Personal computer**					
Yes	224 (71.8%)	88 (28.2%)	1.8 (1.1, 2.9)	**1.7 (1.1, 2.9)**	0.02
No	57 (58.2%)	41 (41.8%)	1.0		
**Computer skill**					
Adequate	186 (75.9%)	59 (24.1%)	2.3 (1.5, 3.6)	1.9 (0.85, 3.4)	0.025
Not adequate	95 (57.6%)	70 (42.4%)	1.0		
**Computer literacy**					
Adequate	189 (78.8%)	51 (21.2%)	3.1 (2.0,4.8)	**2.9 (1.8, 4.6)**	0.000
Not adequate	92 (54.1%)	78 (54.9%)	1.00		
**Computer Training**					
Yes	136 (81%)	32 (19%)	2.8 (1.8,4.5)	**2.0 (1.2, 3.3)**	0.006
No	145 (59.9%)	97 (40.1%)	1.0		
**Internet access**					
Yes	194 (77.3%)	57 (22.7%)	2.8 (1.8,4.3)	**2.1 (1.3, 3.4)**	0.002
No	87 (54.7%)	72 (45.3%)	1.0		

Computer literacy was found positively associated with knowledge .study subjects who had good compute literacy were 2.9 times more likely to be knowledgeable about Telemedicine than their equivalents (AOR = 2.9; % CI: [1.8, 4.6]).

Participants have received computer training were about 2.0 times more probable to be knowledgeable about Telemedicine than their equivalents (AOR = 2.0; % CI: [1.2, 3.3]).

## Discussion

The primary participants in telemedicine adoption are health care providers, who are expected to be more knowledgeable than others. According to the findings of our research, respondents have a better understanding of telemedicine.

In this study, the majority of health professionals had good knowledge of telemedicine. This finding is significantly higher than that of another Ethiopian study (12).

This substantial difference might be due to sample size, study area and more than study subjects at private hospitals in this study have access to the internet and computer. In contrast, this finding is lower than studies done (17, 18). This significant difference may be due to differences in information communication infrastructure, and socio-economic differences.

We found that own personal computer was positively associated with knowledge of telemedicine. Participants who own personal computers were 1.7 times more likely to know about telemedicine compared to their counterparts. This finding is consistent with a study conducted (19).

Computer training was found significantly associated with knowledge of telemedicine. Those study subjects who took computer training were 2.0 times more likely to have knowledge of telemedicine compared with those who did not take it. The result is consistent with studies conducted on health professionals' knowledge and attitude towards telemedicine (20–22). A possible reason for this could be computer training is more likely to increase participant familiarity with using technologies. Additionally, the explanation might be training and education usually changes people's views, and upgrade knowledge levels, and perceptions. Knowing the updated technology passionate for upcoming in their institution.

Computer literacy was found positively associated with knowledge of telemedicine. In this study, those computer-literate health professionals were 2.9 times more likely knowledgeable than their counterparts. This is in line with studies done (21, 23).

The possible explanation might be knowing how to use computer technologies in day-to-day activities increase to use of advanced technologies.

Internet availability was found positively associated with knowledge of telemedicine. Health professionals having Internet availability in the workplace were 2.1more likely to have good knowledge than equivalents. This is consistent with studies done in (24–26). This might be because the internet influences access how new advanced technologies applications in the health system. Internet exposure can impact humankind's daily life.

## Conclusion and recommendations

In general, nearly two-thirds of health professionals know telemedicine Variables including having a laptop, computer literacy, computer training, and internet availability at workplace were the significant factors for knowledge. These findings indicate that providing training for health professionals to lay the base for the fruitful adoption of Telemedicine systems in limited resource settings.

## Data Availability

The raw data supporting the conclusions of this article will be made available by the authors, without undue reservation.

## Appendix

**Part 1: socio-demographic variables.**

(Please encircle your answer)

**Table:**

No.	Question	Response Option	Code	Skip
101	Sex	1. Male		
		2. Female		
102	Age	______ In a year		
103	Professions	1. Medical Doctor		
		2. Health Officer		
		3. Nurse		
		4. Midwifery		
		5. Pharmacy		
		6. Medical Laboratory		
		7. Radiologist		
		8. physiotherapy		
		9. Anesthesia		
		10. Optometry		
		11. Psychiatry		
		12. Other _______		
104	Educational Status	1. Diploma		
		2. Bachelor Degree		
		3. General Practitioners (GP)		
		4. Specialty (MD+)		
		5. Master's Degree		
105	What is your work experience (years)?	_______In Years		
106	Do you have your own Computer/laptop	1. Yes		
		2. No		
107	Do you have your Smartphone	1. Yes		
		2. No		

**Part 2: Technical factors related questions** (27, 28)

**Table:**

**Instruction: *Please try to answer questions by encircling the number* that best reflects your computer skill 1= Strongly Disagree 2 = Disagree 3 = Neutral 4 = Agree 5 = Strongly Agree**						
201	Can you properly turn on and shut down a computer by yourself?	1	2	3	4	5
202	Are you able to manipulate basic Microsoft Office, Microsoft Office Excel, and Microsoft Office PowerPoint by yourself?	1	2	3	4	5
203	Are you able to search, download information from The internet by yourself?	1	2	3	4	5
204	Can you manipulate Cut, Copy, Past digital data Like Text, Images, Audio, and Video by yourself?	1	2	3	4	5
205	Have you able to send digital data like text images, video audio?	1	2	3	4	5
**For each of the questions below, please circle the number that best reflects your Computer literacy 1 = Strongly Disagree, 2 = Disagree, 3 = Neutral, Agree, 4 = Agree, 5 = Strongly Agree**						
206	I am interested in working with computers	1	2	3	4	5
207	I have moderate skill in using computers	1	2	3	4	5
208	I think that healthcare delivery can be improved by using computers	1	2	3	4	5
209	I feel that using computers will support me to be more efficient in the future	1	2	3	4	5
2010	I think that computers are easy to use.	1	2	3	4	5

**Part 3: Organizational Factors Related Question** (29)

**Table:**

301	Is their computer available at your office	1. Yes
		2. No
302	Have you taken any computer training that helps with Telemedicine implementation?	1. Yes
		2. No
303	Do you think you have internet access in your office?	1. Yes
		2. No
304	Do you think that your organization has IT support staff?	1. Yes
		2. No
305	Is there stand by a generator in your organization	1. Yes
		2. No

**Part 4: Knowledge towards Telemedicine** (21, 28)

**Table:**

**Instruction: Please try to answer questions by encircling the number that reflects your level of agreement on Telemedicine knowledge. 1. Yes, 2. No**		
No.	Question	Response question
401	Have you ever heard about Telemedicine ?	1. Yes
		2. No
402	Telemedicine means providing health care services at a distance by using telecommunication technology to provide medical information and health services?	1. Yes
		2. No
403	Online interaction between patients and doctors is possible through a Telemedicine system?	1. Yes
		2. No
404	Patients’ examination and investigation can be communicated through a Telemedicine system?	1. Yes
		2. No
405	Electronic medical records of patients’ registration can be maintained through Telemedicine?	1. Yes
		2. No
406	Telemedicine can be used in a public health emergency, prison facility, and schools for delivering health services?	1. Yes
		2. No
407	Telemedicine service can reduce unnecessary referrals and transportation costs?	1. Yes
		2. No
408	There are different Telemedicine applications like Tele-radiology teleconsultation, Telesurgery, Tele-conferencing, and so on?	1. Yes
		2. No
409	Telemedicine system is important on public health issues like chronic disease management and communicable and non-communicable?	1. Yes
		2. No
410	Computer technology, Telecommunication technology / Health care technology can be used in Telemedicine practice?	1. Yes
		2. No
411	Store / Forward method is an approach to delivering Telemedicine services through email?	1. Yes
		2. No
412	The real-time/live conference method is an online approach to deliver Telemedicine services?	1. Yes
		2. No
413	Telemedicine application is important for remote patient care and management especially elders and disabled?	1. Yes
		2. No
414	Telemedicine is relevant to educate patients and health professionals on managing health problems?	1. Yes
		2. No
415	There are different Telemedicine application areas in clinical practice like radiology, pathology, surgery… etc.?	1. Yes
		2. No
416	Telemedicine can improve the quality and accessibility of health care services?	1. Yes
		2. No
417	We can consulates with a senior physician through video conferencing?	1. Yes
		2. No
418	The remote diagnosis and treatment of a patient through telecommunication technology?	1. Yes
		2. No
